# Response: Commentary: Metacognition and Perspective-Taking in Alzheimer's Disease: A Mini-Review

**DOI:** 10.3389/fpsyg.2020.00453

**Published:** 2020-03-24

**Authors:** Elodie Bertrand, Anna Fischer, Daniel C. Mograbi

**Affiliations:** ^1^Department of Psychology, Pontifical Catholic University of Rio de Janeiro, Rio de Janeiro, Brazil; ^2^Department of Psychology, Universidade Do Grande Rio (Unigranrio), Duque de Caxias, Brazil; ^3^Department of Psychology, Institute of Psychiatry, Psychology & Neuroscience, King's College London, London, United Kingdom

**Keywords:** metacognition, self-awareness, anosognosia, dementia, Alzheimer's disease

## Introduction

Impaired self-awareness is a frequent characteristic of dementia, particularly Alzheimer's disease (AD; Morris and Mograbi, 2013), leading to various negative consequences for the patients and their caregivers, such as reduced treatment adherence and higher caregiver burden (Seltzer et al., 1997; Patel and Prince, 2001; Bertrand et al., 2013). Lack of self-awareness is a complex phenomenon and its clinical presentation is heterogeneous (Clare et al., 2005). For example, it has been shown that people with AD (PwAD) may present impaired self-awareness for some deficits, but preserved awareness of other difficulties (Vasterling et al., 1995; Verhülsdonk et al., 2013; Bertrand et al., 2019). The Cognitive Awareness Model (CAM; Agnew and Morris, 1998; Morris and Hannesdottir, 2004; Hannesdottir and Morris, 2007; Morris and Mograbi, 2013) provides a neurocognitive explanation of lack of self-awareness, trying to account for the complexity of this concept.

Based on the framework developed by Stuss and Anderson (2004) to understand the structure of consciousness, Morese et al. (2018) pointed to the differences between the notions of anosognosia and self-awareness. The authors explain that awareness follows a hierarchical organization in which anosognosia is related to lower levels and self-awareness to higher levels. Whilst we agree that self-awareness and anosognosia can be distinguished (Mograbi and Morris, 2018), our view about the relationship between these concepts differs in relation to that proposed by Morese and colleagues.

The term impaired self-awareness has been employed in the seminal work of Prigatano to describe how awareness about self-ability, personal characteristics, and self-performance can be impaired in clinical populations (Prigatano, 2005). We believe that self-awareness refers to, as the term implies, awareness processes that take the self as an object (Morin, 2006), and it can be understood in even broader terms than those suggested by Prigatano, potentially also involving processes linked to knowledge about internal states (interoception), body ownership (proprioception, agency), identity (autobiographical memory), and monitoring/regulatory processes (metacognition, emotional regulation).

Anosognosia refers to awareness of having a condition (and, by extension, its symptoms and consequences; Mograbi and Morris, 2018), not necessarily being linked to lower-order processes. In fact, it is likely that awareness about having a condition is influenced by higher-order factors such as culture (Mograbi et al., 2012, 2015), illness representations (Mograbi et al., 2012) and premorbid personality (Gilleen et al., 2012). It is possible that impairments in different self-awareness processes may lead to anosognosia (or loss of insight, as employed in the psychiatric literature) in clinical conditions. For example, impaired autobiographical memory may be linked to anosognosia in AD (Mograbi et al., 2009), whilst alterations in sense of agency may explain loss of insight in psychosis (Lysaker and Lysaker, 2010). As indicated above, metacognition can be seen as a self-awareness process, and its impairments may lead to specific forms of anosognosia.

## CAM and Executive Anosognosia

Empirical evidence has highlighted an association between frontal lobe dysfunction and reduced self-awareness in AD, both in studies using neuroimaging (Rosen et al., 2010; Zamboni et al., 2013) and studies employing neuropsychological tests of executive functions (Perrotin et al., 2008; Shaked et al., 2014). These data support the idea of a form of anosognosia linked to executive processes (executive anosognosia; Morris and Mograbi, 2013). In the CAM model, comparator mechanisms are responsible for monitoring of performance, comparing the actual performance with previous information about ability stored in a personal data base. The result of this comparison is passed to the metacognitive awareness system, leading to accurate self-awareness. So PwAD might present reduced self-awareness because the comparator mechanisms fail to detect the mismatches between the expected and the current experience. In other words, executive anosognosia, as proposed in the CAM, highlights the association between metacognition and anosognosia, with a deficit in metacognitive abilities leading to anosognosia.

## Apathy and Error Monitoring

Error monitoring is a prerequisite to develop awareness of performance. There is an association between error awareness and apathy in AD, with higher levels of the latter being related to poorer awareness in people with mild AD and MCI (Jacus, 2017). It is possible that this relationship reflects the importance of emotional processing in error monitoring (Mograbi and Morris, 2014). Emotional reactions mark instances of failed task performance with a level of personal significance, and the absence or diminution of error signals caused by apathy could thus be a leading cause of anosognosia in patients with neurodegenerative diseases, by preventing them to consider these events when evaluating their abilities (Rosen, 2011). In addition, apathy and anosognosia rely on shared neural networks. The anterior cingulate cortex (ACC) is a possible neural correlate of both phenomena, since it has been shown that cortical gray matter atrophy in the bilateral ACC is related to apathy severity in PwAD (Marshall et al., 2006, 2007). Furthermore, this region is the most likely generator of error related potentials like the error related negativity (ERN; Van Veen and Carter, 2002). Error-related activity has also been shown in limbic structures (Polli et al., 2009; Pourtois et al., 2010), suggesting that the amygdala may register motivational significance of motor actions, and the dorsal ACC could provide signals related to failure of cognitive control and behavioral adjustment (Pourtois et al., 2010).

## Conclusion

In summary, apparently similar presentations of anosognosia, unawareness of having a condition and its consequences, can be linked to different impairments in self-awareness. For example, difficulties in metacognition may prevent detecting mismatches between expected and current performance, or impaired emotional processing may deprive errors of their affective signature, leading to limited awareness about performance and condition. This is precisely the notion described in the CAM, which tries to deal with heterogeneity of anosognosia in clinical groups, suggesting factors such as memory, executive functions, and top-down/bottom-up modulatory processes that can cause different forms of anosognosia. Future research should explore which self-awareness processes are particularly relevant in the context of specific conditions, investigating the relationship between different self-awareness components and how these relate to awareness of condition, considering the impact of the latter in clinical management of patients.

## Author Contributions

All authors listed have made a substantial, direct and intellectual contribution to the work, and approved it for publication.

### Conflict of Interest

The authors declare that the research was conducted in the absence of any commercial or financial relationships that could be construed as a potential conflict of interest.
